# Camouflage and Clutch Survival in Plovers and Terns

**DOI:** 10.1038/srep32059

**Published:** 2016-09-12

**Authors:** Mary Caswell Stoddard, Krisztina Kupán, Harold N. Eyster, Wendoly Rojas-Abreu, Medardo Cruz-López, Martín Alejandro Serrano-Meneses, Clemens Küpper

**Affiliations:** 1 Department of Ecology and Evolutionary Biology, Princeton University, Princeton, NJ 08544, USA; 2 Museum of Comparative Zoology, Department of Organismic and Evolutionary Biology, Harvard University, Cambridge, MA 02138, USA; 3 Institute of Zoology, University of Graz, Universitätsplatz 2, 8010 Graz, Austria; 4 Laboratorio de Biología Evolutiva, Centro Tlaxcala de Biología de la Conducta, Universidad Autónoma de Tlaxcala, Carretera Tlaxcala-Puebla Km. 1.5, 90070, Tlaxcala, México; 5 Posgrado en Ciencias del Mar y Limnología, Universidad Nacional Autónoma de México, Unidad Académica Mazatlán, Av. Joel Montes Camarena S/N, 82040, Mazatlán, Sinaloa, México; 6 Posgrado en Ciencias Biológicas, Universidad Autónoma de Tlaxcala, México

## Abstract

Animals achieve camouflage through a variety of mechanisms, of which background matching and disruptive coloration are likely the most common. Although many studies have investigated camouflage mechanisms using artificial stimuli and in lab experiments, less work has addressed camouflage in the wild. Here we examine egg camouflage in clutches laid by ground-nesting Snowy Plovers *Charadrius nivosus* and Least Terns *Sternula antillarum* breeding in mixed aggregations at Bahía de Ceuta, Sinaloa, Mexico. We obtained digital images of clutches laid by both species. We then calibrated the images and used custom computer software and edge detection algorithms to quantify measures related to three potential camouflage mechanisms: pattern complexity matching, disruptive effects and background color matching. Based on our image analyses, Snowy Plover clutches, in general, appeared to be more camouflaged than Least Tern clutches. Snowy Plover clutches also survived better than Least Tern clutches. Unexpectedly, variation in clutch survival was not explained by any measure of egg camouflage in either species. We conclude that measures of egg camouflage are poor predictors of clutch survival in this population. The behavior of the incubating parents may also affect clutch predation. Determining the significance of egg camouflage requires further testing using visual models and behavioral experiments.

The appearance of bird eggs has long been of interest to evolutionary biologists, including Darwin^1^, Wallace^2^, Poulton^3^, Cott^4^ and Lack^5^ – and it continues to be the source of active research effort^6^,^7^. The egg phenotype is likely affected by a suite of selective forces related to the thermoregulatory, structural and signaling functions of coloration^6^. From a broad comparative perspective, selection by predators appears to be the primary driver of variation in egg appearance^6^. However, studies at the genus and species levels have yielded mixed support for the idea that egg camouflage confers a survival advantage at the nest^7^. One explanation for this may be that few studies have comprehensively tested 1) the mechanisms by which eggs are camouflaged in wild populations, and 2) the efficacy of those mechanisms in reducing predation. First, the majority of work to date has focused simply on the color of eggs and the background substrate^8^ rather than on more complex aspects of spatial patterning. In fact, the pigmented speckles and markings on eggs (maculation) and the textures in the background substrate also play an important role in camouflage. Second, few studies have assessed how measures of egg appearance directly relate to clutch survival^9^,^10^,^11^,^12^,^13^,^14^,^15^,^16^,^17^. Of these studies, only three quantitatively assessed spatial aspects of egg markings or background substrate textures^11^,^16^,^17^.

Theoretical and experimental work on camouflage has surged in recent years^18^. Evidence from diverse animal systems has shown that organisms can disguise themselves through a variety of mechanisms, including background matching and disruptive coloration^19^. In background matching, an organism matches the coloration or patterning of the background substrate^20^. In disruptive coloration, highly contrasting patterns at the edge of the organism serve to break up its outline^4^,^18^,^21^,^22^. Another way in which organisms can enhance camouflage is by resting on a complex background substrate, which should make search tasks more difficult for predators^23^. To investigate these different forms of camouflage in natural systems, digital photography and image analysis are being rapidly adopted^21^,^24^,^25^. This approach lends new quantitative power to the assessment of camouflage because images capture entire scenes (containing information about the color and patterning of the organism and its background) and can be combined with image analysis techniques, like edge detection algorithms. Investigating pattern and texture in this way can shed light on the perceptual organization of predators^26^.

Two recent studies – in quail^24^ and moths^25^ – have explicitly evaluated different camouflage mechanisms by quantifying aspects of background matching and disruptive coloration in the context of behavior. A third study in nightjars, plovers and coursers assessed plumage and egg camouflage by quantifying color, luminance and pattern variation in the vicinity of the nest^17^. Lovell *et al.*^24^ demonstrated that Japanese Quail (*Conturnix japonica*) choose to lay their eggs in microhabitats that enhance camouflage for their individual egg phenotypes. Females with heavily patterned (maculated) eggs laid their eggs on dark background substrates, probably concealing their eggs by disruptive coloration. Females with only lightly maculated eggs tended to lay their eggs on a background substrate that was a good match to the egg’s background color, probably concealing their eggs by background matching. In quail, and potentially in other avian species^9^,^12^,^14^, individuals can enhance their camouflage by microhabitat selection – or, as in Blue-footed Boobies (*Sula nebouxii*), by actively improving their eggs’ appearance through behavioral modification^27^. Kang *et al.*^25^ identified the concealing mechanisms used by two bark-resting moth species (*Hypomecis roboraria* and *Jankowskia fuscaria*) that reorient their bodies after landing on the bark of a tree. Both moth species shifted their bodies to positions that reduced detection; however, each species exploited different camouflage mechanisms, providing support for the hypothesis that multiple forms of camouflage can be important for concealment. As with quail^24^, moths can overcome constraints on their fixed appearance by modifying their behavior in a way that improves camouflage.

The eggs of ground-nesting birds are a compelling system for investigating mechanisms of camouflage outside of the lab for at least three reasons. First, in open-nesting birds, clutch predation is a major selective force limiting reproductive success^28^. Egg coloration and patterning that increase clutch camouflage to prevent predator detection are generally thought to be important defense mechanisms in these species, though this idea has received mixed support^8^. Second, it is relatively straightforward to photograph clutches of eggs in their natural habitats and to document clutch survival; therefore egg camouflage can be assessed in wild populations, free of artificial stimuli or lab settings. Third, different species of ground-nesting birds often breed in mixed colonies or semi-colonial aggregations, where they are likely subjected to similar selection pressures. Therefore, comparing different characteristics of the eggs allows us to study whether different species rely on the same or different camouflage mechanisms.

To date, plovers and terns have been the subject of several studies on egg camouflage, presenting mixed evidence as to whether aspects of egg appearance and microhabitat affect clutch survival^12^,^14^,^15^,^29^. However, these studies, as with most other field studies of egg camouflage, have not fully quantified and tested the effects of different camouflage mechanisms in a quantitative framework. Usually this is because studies (i) assess only limited aspects of egg camouflage, (ii) rely on a subjective human description of egg camouflage, or (iii) fail to correct for different ambient conditions such as variable light environments. An exception to this is the recent study by Troscianko *et al.*^17^, which examined plumage and egg camouflage in nine species of ground-nesting nightjars, plovers and coursers, using a calibrated camera, visual models for three types of predators (two mammalian, one avian), and a range of quantitative tools. With respect to plover and courser egg camouflage, only contrast (luminance variation) within the eggs and background substrate affected clutch survival: there was no effect of background color matching or pattern matching.

Here, we use digital image analysis and edge detection algorithms to quantify and compare potential egg camouflage mechanisms used by Snowy Plovers and Least Terns breeding at Bahía de Ceuta, Sinaloa, Mexico. We focus on three camouflage mechanisms that fall into two broad classes of camouflage: background matching and disruptive coloration. For each clutch, we assess (i) the degree of pattern complexity matching (a form of background matching), (ii) the degree of disruptive effects (a form of disruptive coloration), and (iii) the degree of background color matching (a form of background matching).

Our study has three main goals. First, we use image analysis to quantify and compare camouflage metrics, testing for differences between plovers and terns. Second, we test whether tern and plover nests differ in their survival. Third, we determine whether any of the camouflage mechanisms has an effect on clutch survival by examining daily survival rates of clutches.

## Methods

### Field Procedure

**Figure 1:**
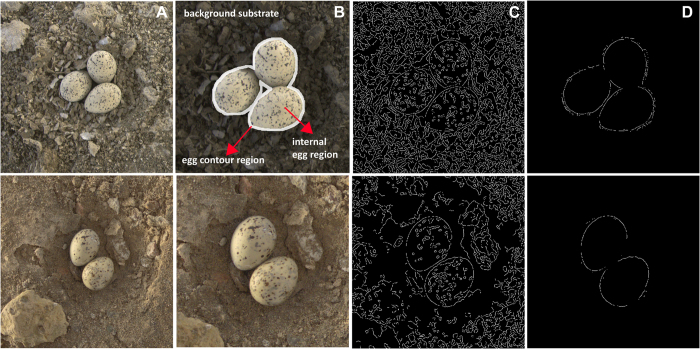
(**A**) Representative Snowy Plover (top row) and Least Tern clutch (bottom row) at Bahía de Ceuta. All images were analyzed at the same scale. (**B**) We separated images into three regions: the background substrate, the internal egg region and the egg contour region. The image in the top row shows the background substrate darkened (for effect only) and the egg contour region highlighted (for effect only). Here we show the images zoomed in, for clarity. (**C**) Edges in the eggs and in the background substrate are identified using an edge detection algorithm. (**D**) Edges in the egg contour region are identified and quantified. This Snowy Plover clutch was laid on a more complex background substrate (more edges) and had a greater number of detectable edges in the egg contour region, compared to this Least Tern clutch. Egg images by Wendoly Rojas-Abreu.

We carried out fieldwork at Bahía de Ceuta, Sinaloa, Mexico (23° 54′ N, 106° 57′ W) from April to June 2012. Snowy Plovers and Least Terns nest together in mixed semi-colonial aggregations at salt flats surrounded by mangrove forests once the tidal water has retreated and ground becomes available for nesting. About 30–100 pairs of Snowy Plovers and 200–300 pairs of Least Tern use the salt flats for reproduction during spring and summer annually^30^. Snowy Plovers typically lay three-egg clutches whereas Least Tern clutches typically contain two eggs. Egg sizes and patterns are similar to a human observer (Fig. 1). We searched for nests using a mobile blind or by car (details in ref. 31) and identified parents according to typical incubation postures. Once we found a nest, we estimated the onset of incubation and hatching date by floating the eggs in lukewarm fresh water, assuming an incubation period of 25 days for both species. We also measured the width and length of the eggs (to the nearest 0.1 mm) and recorded the geographic position of the nest with a handheld GPS (to the nearest 3 m). Snowy Plover parents were individually marked with color and metal bands as part of an ongoing long-term study into the evolutionary ecology of breeding behavior^32^. For Least Terns, parents were unmarked, but all surveyed nests were found within a period of 15 days. The incubation start date of the last nest coincided with the date of the first nest failure. Since Least Terns parents are monogamous and look after only one nest at a time^33^, we are certain that each nest was produced by a different pair.

We took pictures of completed clutches (*i*.*e*., those containing the modal egg number or with the onset of incubation >5 days back) during morning hours between 6:30 and 9:00 a.m. by placing the mobile blind above the clutch to ameliorate effects of variable light conditions. We took three pictures from approximately 1 m above the ground using a Canon EOS 1100D with the lens (18–55 mm) at 45 mm, with the white balance set to ‘cloudy’ and a ColorChecker Card (X-Rite, Grand Rapids, MI, USA) placed next to the nest. All images were stored in RAW (.CR2) file format.

Clutches were checked regularly for survival every three to four days to determine clutch survival. Once the clutch reached an age of 20 days, eggs were checked every other day for noises from the chicks and/or pipping. When these were detected, we checked clutches daily for hatching. Possible reasons for clutch failure were ‘parental abandonment’ – parents abort incubation for unknown reason, ‘human-induced destruction’ – by grazing cattle or cars, ‘flooding’ – clutch lost to rain or tidal water, ‘unhatched’ – clutches incubated for more than 30 days but no chicks hatched, or ‘predation’– eggs disappeared between control visits and other reasons for failure did not apply. Suspected clutch predators of plover and tern nests based on tracks near predated nests or observations at Bahía de Ceuta include Coyote (*Canis latrans*), feral dogs (*Canis familiaris*), Raccoon (*Procyon lotor)*, Bobcat (*Lynx rufus*), Opossum (*Didelphis virginiana*) and Crested Caracara (*Caracara cheriway*).

Fieldwork permits (02898/12) were granted by the Secretaría de Medio Ambiente y Recursos Naturales (SEMARNAT) to Lydia Lozano-Angulo, Martín Alejandro Serrano-Meneses, Medardo Cruz-Lopéz and Clemens Küpper. Our study was performed in accordance with the approved guidelines outlined by SEMARNAT, and all experimental protocols were approved by the Ethics Committee of the Universidad Autónoma de Tlaxcala.

### Image Calibration

After converting the RAW images to TIFF files, we used the PictoColor inCamera 4.5 plug-in for Photoshop CS6, which automatically equalizes and linearizes each image^34^,^35^,^36^. The inCamera plug-in creates a custom color profile that adjusts the colors in the image based on the standardized color levels contained in the ColorChecker card. We assigned and converted each image to its respective custom profile. Using the Matlab Image Processing Toolbox (Mathworks, Natick, MA), we imported the images and converted them to the CIELAB color space (CIE, 1976). This step required first converting the input images, which were in the Adobe RGB 1998 color space, to the CIE XYZ color space; after this, images were converted to the CIELAB color space. We cropped all images to a region approximately equal to the size of 13.3 by 13.3 centimeters, with the clutch of eggs positioned at the center of region of interest. Clutches contained 2–3 eggs.

Subsequent analyses were performed on the images after conversion to the CIELAB color space. The CIELAB color space is a perceptually uniform color space designed to provide estimates of human luminance and chromatic perception. Since the composition of the local egg predator community at Bahía de Ceuta is unknown, we elected to use the CIELAB space, which yields device-independent measures, permits comparison to other recent studies of egg camouflage (Lovell *et al.*^24^) and provides a first approximation of camouflage metrics. However, future studies in this system should incorporate visual models that are relevant to the target signal receivers^7^,^17^,^37^,^38^, which likely include mammals, non-avian reptiles and birds (see Discussion).

### Image Analysis

To quantify aspects of camouflage, we used methods similar to those detailed in recent studies by Kang *et al.*^25^ and Lovell *et al.*^24^ and, where possible, we apply the same variable names. First, we separated the image into three different sections: the egg contour region (containing the region near the outer edges of the clutch, calculated by eroding the outline of the egg region by 4 pixels and dilating it by 8 pixels, to create a 12 pixel-wide band), the internal egg region (containing the internal area of the clutch of eggs, after the outline of the egg region was eroded by 4 pixels), and the background substrate region (the ground substrate surrounding the clutch, beyond the dilated egg region outline) (Fig. 1). The eggs consisted of a background color (hereafter ‘egg background color’) and pigmented splotches, speckles and markings (hereafter ‘egg maculation’). Next, we quantified variables that relate to different forms of camouflage: pattern complexity matching, disruptive effects, and background color matching. In CIELAB color space, images are processed in three channels, where the first represents luminance (L) and the latter two (a and b) represent color variation along the green-red and blue-yellow axes, respectively. For most animals, texture and pattern processing involves the use of achromatic (luminance) signals^39^. For our analyses involving pattern complexity matching and disruptive effects, we used the CIELAB luminance (L) image only.

All analyses were performed in MATLAB (MathWorks, Natick, MA, USA) using custom code and modified functions from the MathWorks Central File Exchange (*e*.*g*., “DeltaE”, Image Analyst 2011). We have compiled a MATLAB GUI with functions for edge detection called EdgeDetector, which is available upon request.

### Quantifying pattern complexity matching

One way in which animals can achieve camouflage involves resting on a substrate with high visual complexity; this can enhance camouflage irrespective of the organism’s own pattern^23^. Furthermore, an organism can enhance its camouflage if its pattern matches the pattern of the background^40^. To measure the degree of background complexity, we calculated the proportion of edges detected in the background substrate region (**BgEdge**, the number of edge pixels detected in the background substrate/the total number of pixels in the background substrate)^25^. To measure the degree to which the egg maculation matched the complexity of the background substrate patterns, we calculated complexity ratio (**CompRat**)^25^, which is the ratio of the proportion of edges detected inside the eggs (**EggEdge**) to the proportion of edges detected in the background (**BgEdge**). In theory, when CompRat is close to 1, the organism achieves better pattern complexity matching. To detect edges, we used the MATLAB Image Processing Toolbox to apply the Canny edge detection algorithm to the images (settings: threshold = 0.2 and sigma = 3)^24^. The Canny edge detector is a common computer vision algorithm that locates edges in a scene by searching for local maxima of the intensity gradient of the image^41^. Though most work on the visual encoding of edges has been based on humans^42^, other vertebrate visual systems may detect edges in a similar way (*e*.*g*., in pigeons^43^,^44^).

Our use of edge detection algorithms for quantifying pattern complexity and disruptive markings (next section) differs from some methods used elsewhere to describe the patterns on eggs, including granularity analysis^45^, spatial frequency comparisons across a visual scene^17^, and pattern recognition algorithms^46^. All of these methods are valuable depending on the question at hand. Edge detection algorithms are convenient because they help reveal which camouflage mechanism – background matching or disruptive coloration – may be responsible for reducing the detection of egg edges^24^. Although eggs, unlike two-dimensional moths (Kang *et al.*^25^), are round and therefore lack clearly defined contours (*i*.*e*., the perceived outline of the egg will change based on the receiver’s viewing angle), we assume that the predators are viewing eggs from above the nest scrape and therefore edge detection will be of biological significance.

### Quantifying disruptive markings

Another way in which organisms can achieve camouflage is by hiding their edges using disruptive markings. This can be achieved by differential blending, when the outline of the egg blends into the color of the background, obscuring the egg’s true edges. We calculated the proportion of edges in the egg contour region, which is equal to the edge pixels in the egg’s contour region/total number of pixels in the egg contour region (**ContEdge**)^25^. Another form of disruptive markings occurs when the internal patterns also make it difficult to detect the organism’s outline. We therefore calculated the Disruptive Ratio (**DisRat**)^25^, which is the ratio between ContEdge and EggEdge. When the ratio is close to 1, the edges in the contour should be similar to the edges in the eggs themselves. When the ratio is less than 1, the edges in the contour are harder to detect than those in the eggs, leading to low detectability. When the ratio is greater than 1, the edges in the contour are easier to detect than those in the eggs, leading to high detectability. Finally, we calculated the Visibility Ratio (**VisRat**), which is a measure of the detectability of the contour edges relative to the edges in the background substrate. Our measure is similar to that calculated in Lovell^24^ except that we use the proportion of edge pixels in the contour/the proportion of edge pixels in the background substrate (ContEdge/BgEdge), rather than the absolute edge pixels in the contour/absolute edge pixels in the background substrate. This normalization is important because the contour and background sections of the images will differ in pixel size from one image to the next. In theory, higher VisRat leads to higher detectability of the eggs’ edges against the background substrate. As described in the previous section, we used luminance images for these assessments, and we used the Canny edge detection algorithm to detect edges in the different image regions.

### Quantifying background color matching

We calculated LAB values of the egg background color, the egg maculation color and the background substrate color. We also calculated the mean color of the clutch of eggs, which included the egg background and egg maculation regions together. We then calculated **DeltaE**, the Euclidean distance between mean egg color and the mean background substrate color in the CIELAB space. However, the DeltaE measure does not account for the heterogeneity of color in the background substrate. To overcome this, we included an additional measure, the proportion of pixels in the background substrate that matched – within one standard deviation – the average color of the clutch (**PropBgToEggColorMatch**). Theory predicts that color matching enhances camouflage: it is an important component of background matching. We therefore predicted that low values of DeltaE and high values of PropBgToEggColorMatch would be associated with enhanced camouflage. Using eggs clutches at the Museum of Comparative Zoology (Harvard University, Cambridge, MA), we measured reflectance spectra from eggs in 4 Snowy Plover and 3 Least Tern clutches to test for the presence of ultraviolet signals on eggs.

### Statistical analyses

We compared pattern complexity (BgEdge, EggEdge, CompRat), disruptive effects (ContEdge, DisRat, VisRat) and characteristics of the color of eggs and background substrate (LAB of egg background substrate, LAB of mean color of eggs, LAB of egg maculation, proportion of maculation, DeltaE and PropBgToEggColorMatch) between the two species using t-tests, adjusting p-value thresholds to control for multiple testing^47^. For each variable, we assessed homogeneity of variance with Levene’s test and normality with Shapiro-Wilk test. Variables ‘a’ of mean egg color, ‘a’ of egg background, ‘a’ of egg maculation, DeltaE, DisRat and CompRat were log transformed to achieve better fits to normal distributions. We used Cox models (R package ‘survival’) to model species differences and the impact of egg camouflage on survival during the full incubation period. Predation was the terminal event in these models. All other fates were censored events, and we took the last observation when the clutch still contained eggs and was attended by the parents as the final observation.

We assessed the effect of camouflage variables on survival using an information theoretic approach^48^, comparing a suite of simple and complex candidate models. The simplest model contained only **Species** fitted as a fixed factor. This model served as our null hypothesis, *i*.*e*., there is no effect of any camouflage variable on survival in either species. We tested the main predictors of egg camouflage (DisRat, VisRat, CompRat, DeltaE, BgEdge, EggEdge, ContEdge and PropBgToEggColorMatch) in a series of models. We tested for multicollinearity of predictor variables using variance inflation factors and stepwise removed variables with factors larger than 5, dropping the variable with the smallest effect on survival. Because of a significant effect of Species in the initial model (see Results), we prepared for each camouflage predictor three separate models: (i) without Species, (ii) with Species, and (iii) with Species and the interaction between Species and the given camouflage variable. Our interpretation of the results was as follows: (i) if any of the models without Species was the best, then the camouflage variable affected survival in both species but there was no difference in survival between species; (ii) if any of the models including Species was the best, then the camouflage variable affected survival in both species after controlling for Species; (iii) if any model with a species-camouflage interaction was the best, then the camouflage variable affected clutch survival in plovers and terns differently.

We then fitted an additional, more complex model that contained Species and all camouflage predictors that showed significant differences between terns and plovers, leaving out the terms that had shown multicollinearity. The two most complex models included all camouflage variables pruned for multicollinearity (i) without Species or (ii) with Species. Note that for CompRat, we also fitted a quadratic effect of the transformed variable since the best camouflage is achieved when the ratio equals 1.

We ranked models using AIC adjusted for small samples (AICc). This approach assesses the level of support from the data for the current model compared with the most highly ranked model using AICc differences (Δ_i_) and associated model weights (ω_i_) but does not provide p-values^48^. Candidate models with Δ_i_-values ≤2 have substantial support, whereas those with Δ_i_ > 10 have little or no support. Any model for which ω_i_ > 0.9 is chosen as representing the data best. When no model has ω_i_ > 0.9, there is support for multiple candidate models^48^; in this case, we averaged parameter estimates to provide estimates of the strength, direction and uncertainty of parameters. Model ranking and averaging was done with the ‘MuMIn’ package version 1.15.1 in R. We provide hazard ratio (HR) and 95% confidence intervals (CI) for the averaged parameter estimates for the survival models with ∆i ≤ 2.

All statistical analyses were run in R version 3.2.2 (“Fire Safety”, R Development Core Team 2015).

## Results

### Comparing pattern complexity matching

**Figure 2:**
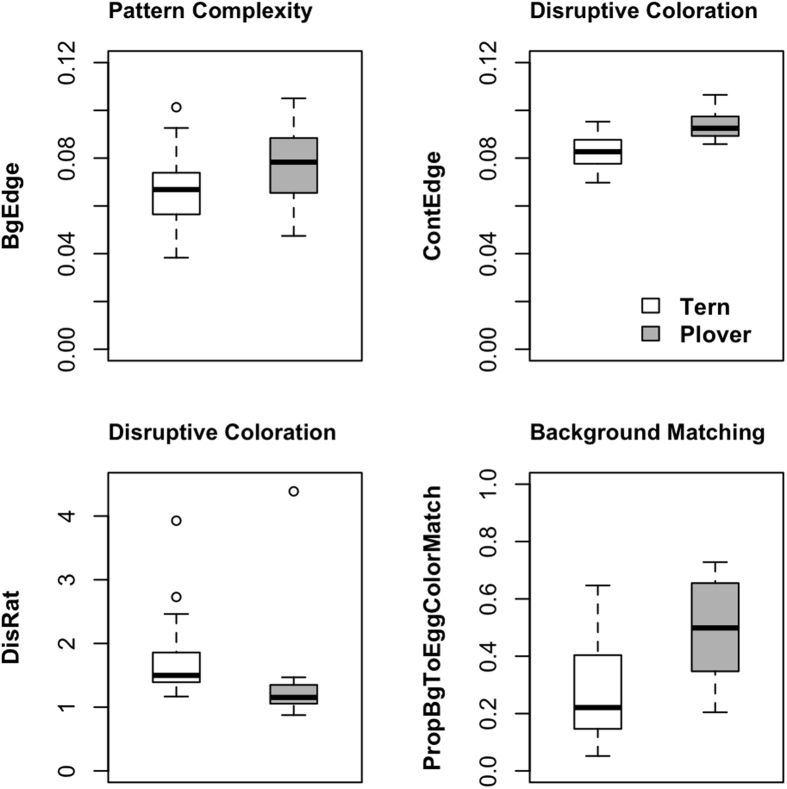
Differences in camouflage mechanisms between Least Tern and Snowy Plover clutches at Bahía de Ceuta. In terms of pattern complexity, Snowy Plovers nested on background substrates with more edges, which likely enhances camouflage. In terms of disruptive coloration, Least Terns and Snowy Plovers appeared to use different strategies to enhance camouflage. Least Terns had less detectable edges in the egg contours, whereas Snowy Plovers had a lower Disruptive Ratio to achieve better camouflage. Snowy Plover clutches had better background color matching than Least Tern clutches. See text for details.

We found that Snowy Plover clutches (N = 30), compared to Least Tern clutches (N = 24), were laid on background substrates with more edges (Table 1, Fig. 2), suggesting better Snowy Plover clutch camouflage based on this metric^49^. We also found that there were more edges in the egg interior of Snowy Plover clutches than in Least Tern clutches (Table 1). The complexity ratio (CompRat), which is the ratio of interior egg edges to edges in the background substrate, was significantly higher for Snowy Plovers than for Least Tern clutches (Table 1). However, there was no significant difference between the quadratic terms (CompRat^2: t = −0.33, df = 41.5, p-value = 0.73), which measure the difference from CompRat = 1, *i*.*e*., the ratio at which the best camouflage is achieved. This indicates that Snowy Plovers and Least Terns do not differ in the extent to which their egg maculation patterns match the complexity of the background substrate.

### Comparing disruptive effects

When we compared the edges in the contour of the clutches (ContEdge), we found that Snowy Plovers had more edges than Least Terns (Table 1, Fig. 2), suggesting that Snowy Plover clutches are more detectable and less well camouflaged than Least Tern clutches based on this metric. However, when we compared the Disruptive Ratio (DisRat), which measures the ratio of edges in the egg contour region (ContEdge) to edges in the egg interior (EggEdge), we found that the Snowy Plover clutches were better camouflaged since they had a significantly lower DisRat (Table 1). Even though both Snowy Plovers and Least Terns had DisRat >1, which indicates that edges in the egg contour region are more easily detected than the internal egg edges, DisRat was lower for Snowy Plovers than for Least Terns. Therefore, Snowy Plover egg outlines are less conspicuous relative to internal egg edges than Least Tern egg outlines. There was no significant difference between the two species in terms of the Visibility Ratio (VisRat), which measures the detectability of the contour edges relative to the edges in the background substrate (Table 1).

### Comparing background color matching

When we considered the background (non-maculated) and maculated regions of eggs, we found no significant difference between Least Tern and Snowy Plover egg color (Supplementary Information Table 1). When we considered the mean color of eggs (background and maculated regions combined), Least Tern and Snowy Plover eggs differed significantly only in terms of ‘a’ (the red-green axis of the CIELAB space), with Least Tern eggs having a higher value (Supplementary Information Table 1), and not in terms of ‘L’ or ‘b’ values (Supplementary Information Table 1). Least Tern and Snowy Plover eggs did not differ in proportion of maculation (Supplementary Information Table 1).

When we compared DeltaE between the Least Tern and Snowy Plover eggs and the substrate, there was no significant difference (Table 1). However, when we compared the proportion of pixels in the substrate that matched the egg clutch, we found that Snowy Plover eggs were a better match to colors in the background (Table 1, Fig. 2). This latter measure captures heterogeneity in the substrate and may be an important component of camouflage^50^.

Our analyses based on representative museum eggs showed that Snowy Plover and Least Tern eggs do reflect a modest amount of ultraviolet light (Supplementary Information Fig. 1), with the degree of UV-reflectance similar between the two species. We did not find evidence of “hidden” maculation patterns reflecting only in the UV.

### Comparing survival between species

**Figure 3:**
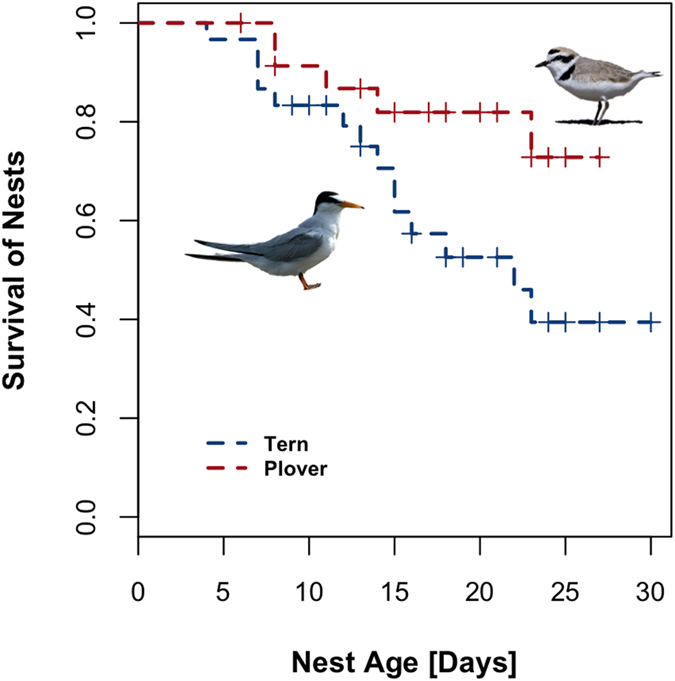
Survival of 24 Least Tern and 30 Snowy Plover nests over the incubation period at Bahía de Ceuta. The image of the Snowy Plover is from Wikimedia Commons^66^ . The image of the Least Tern is from Wikimedia Commons^67^.

Fourteen (58%) Least Tern clutches and five (17%) Snowy Plovers clutches were predated. Least Tern clutches had a 2.9-fold higher risk of being predated than Snowy Plovers when no camouflage variable was considered (Fig. 3: Cox model: HR = 0.35, lower 95% CI: 0.13, upper 95% CI = 1, n = 54, p = 0.049).

Models including BgEdge, ContEdge and EggEdge, in addition to the ratio predictors of camouflage (DisRat, VisRat and CompRat), suffered from multicollinearity (variance inflation factors >5) and therefore we dropped the ~Edge terms from further analyses. In total we compared 19 models. The best 11 candidate model all contained Species as a predictor variable (Table 2). The best model only contained Species as an independent predictor of egg survival (Table 2). However, five more candidate models had Δi-values ≤2 when they included either background color matching (PropBgToEggColorMatch) or a disruptive mechanism (DisRat or CompRat^2) for camouflage. More complex models generally performed poorly in describing clutch survival. Model averaged parameter estimates for the best five models all included a HR of 1 in the 95% CI, indicating no significant effect of the averaged estimates (Species: HR = 0.46, lower 95% CI: 0.04, upper 95% CI: 6, n = 54; DisRat: HR = 7.14, lower 95% CI: 0.37, upper 95% CI: 139, n = 54; CompRat^2: HR = 0.18, lower 95% CI: 0.004, upper 95% CI: 8, n = 54; PropBgToEggColorMatch: HR = 8.09, lower 95% CI: 0.27, upper 95% CI: 244, n = 54).

## Discussion

Egg camouflage appears to be a crucial part of nest defense for ground-nesting birds. However, several important questions remain largely unanswered. Do species appear to use the same or different camouflage mechanisms to conceal their eggs? Which aspects of egg camouflage affect survival? Our analyses revealed that there are several key differences in the putative egg camouflage strategies used by Snowy Plovers and Least Terns, at least from the perspective of a generic predator. However, egg camouflage does not explain the differences in egg survival in this system.

Our first set of findings are in line with those of studies showing that different species might exploit different camouflage mechanisms^25^. In terms of pattern complexity, Snowy Plovers laid their eggs on background substrates with more edges, a strategy that might confer better camouflage by making search tasks more challenging for predators^49^. However, we found that Snowy Plovers and Least Terns were similar in the extent to which their internal egg patterning matched the complexity of the background substrate. In terms of disruptive effects, Snowy Plover eggs had a greater number of detectable edges in the egg contour region, indicating that the outline of Snowy Plover eggs may be more easily detected by a predator. Despite this, Snowy Plovers had a lower Disruptive Ratio than Least Terns, suggesting that Snowy Plover eggs were more camouflaged than Least Tern eggs when we also consider the internal patterning of the eggs. In other words, Snowy Plover egg edges were less conspicuous relative to the egg’s internal edges.

In terms of egg-to-background substrate color matching, Snowy Plover eggs proved to be a better match to the background substrate when we accounted for heterogeneity in the background substrate. When we considered only the mean color of the background substrate, there was no difference in the extent to which Snowy Plover and Least Tern eggs matched the background substrate. Overall, Snowy Plovers nested on more complex background substrates, had eggs with less conspicuous contours relative to internal egg patterning and laid their eggs on a background substrate that was a better match to their egg color. Taken collectively, these results suggest that Snowy Plover eggs tend to be better camouflaged than Least Tern eggs. However, both species likely use a combination of mechanisms, with elements of background matching and disruptive coloration, to improve camouflage.

We also found that Snowy Plover clutches survived better than Least Tern clutches. Only 17% of Snowy Plover nests were predated over the course of the incubation period, compared to 58% of Least Tern clutches. However, in our statistical models, we did not find that any single measure of camouflage was a good predictor of survival. Ultimately, the best statistical model was our null model, in which differences in survival were related to species only, with no additional effect of camouflage variables. Why doesn’t camouflage affect survival? One explanation may be that there are complex statistical interactions that we did not include in our models because we lacked an explicit hypothesis about what those interactions might be. Various predator species may evaluate visual cues differently when searching for eggs (or not at all, if olfactory cues are used instead). Egg predators of the two species include several avian, reptilian and mammalian animals with diverse luminance vision and color vision mechanisms. Most non-primate mammals are dichromats (two color cones), while many snakes are trichromats (three color cones) and birds are tetrachromats (four color cones). If dichromatic predators tend to rely primarily on achromatic cues like pattern and secondarily on chromatic cues when searching for eggs, then there may be interactions between certain achromatic and chromatic camouflage metrics. These relationships might be masked if other predators, like trichromats or tetrachromats, prioritize cues differently. To resolve this, an experimental approach is needed to uncover which features of camouflage are relevant to different predator species.

An alternative explanation may be that lower predation in Snowy Plovers does not directly result from improved egg camouflage. Instead, clutch predation might be related to other factors such as nest location or nest density. Furthermore, the two species may differ in some aspects of their conspicuousness and behavior, which could impact survival. Snowy Plover adults appear, at least to human eyes, to be more camouflaged than Least Terns, which are more conspicuous due to their large, highly contrasting plumage patches. Better adult camouflage can result in reduced nest predation. For example, in Red-capped Plovers (*Charadrius ruficapillus*), parents respond to threats from visually-guided diurnal predators by modifying their incubation behavior^51^. Brightly colored males tend to incubate at night, while duller females incubate during the day; experiments with artificial model adults indicate that the risk of clutch predation increases if males incubate during the day. If Snowy Plover and Least Tern adults vary greatly in the extent to which they are camouflaged, this might explain why we observed differences in clutch survival – if predators use the incubating parents as their main visual cue.

Importantly, Snowy Plovers and Least Terns behave differently to distract predators from the nest^33^,^52^; whereas Snowy Plovers run away when a predator approaches, Least Terns will attack potential predators. This could explain why selection for good egg camouflage might be stronger in Snowy Plovers than in Least Terns, since Snowy Plover eggs are more frequently exposed. There is support for the idea that species differences may be important when determining the extent to which egg camouflage is an effective form of nest protection^53^. For example, in Northern Lapwings (*Vanellus vanellus*) and Little-ringed Plovers (*Charadrius dubius*), larger, more aggressive lapwings often attack predators, and they are far more likely to remain on or near the nest, preferring “fight” to “flight.” Little-ringed Plovers, on the other hand, are smaller and quicker to flee the nest, so they may rely more heavily on egg camouflage^53^. In many cases, egg camouflage is likely to be the last line of defense against predators^7^. Understanding the hierarchy of defenses involved at the nest is an important priority for investigations of egg camouflage (see below).

Even though the best statistical model was our null model, in which only species identity had an effect on survival, the alternative candidate models (Table 2) show that the various camouflage metrics differ in the strength of the effect they have on survival. For example, the Disruptive Ratio (DisRat, the ratio of edges in the egg contour region to edges in the egg interior) did a better job of predicting survival than the Visibility Ratio (VisRat, the detectability of the contour edges relative to the edges in the background substrate). Future studies could test the hypothesis that, in this system, effective disruptive camouflage may have more to do with minimizing the detectability of the eggs’ internal features (DisRat) than with minimizing the detectability of the eggs relative to their background substrates (VisRat).

A recent study assessed egg camouflage in nine species of plovers, coursers and nightjars, using representative visual models for di-, tri-, and tetrachromatic predators^17^. Across these nine species, the authors found that only the contrast of the eggs effected survival; low contrast eggs (low variability in luminance across the egg, *i*.*e*., dark maculation on a dark egg or light maculation on a light egg) survived better than high contrast eggs (high variability in luminance across the eggs, *i*.*e*., dark maculation on a light egg), unless high contrast eggs were laid on high contrast (high variability in luminance) background substrates. However, this result emerged when the nine species were considered together, leaving the extent to which species differences explained nest survival an open question.

We found that Snowy Plovers and Least Terns differ in in camouflage metrics and in nest survival but not nest habitat, since they breed together in a mixed colony and therefore likely have the same egg predators. However, we did not find an effect of a camouflage metric on survival, and we therefore conclude that other differences between the two species account for the differences in predation. Overall, selection for camouflaged eggs may operate differently at the individual, species, genus and family levels. Moving forward it will be important to examine camouflage carefully at each of these taxonomic levels.

There are two main goals for future work in our system. First, it will be critical to describe the suite of local egg predators at Bahía de Ceuta and to model egg camouflage from the perspective of the relevant predator visual systems. The increasing ease with which camera traps and videos can be installed at nest sites^17^,^54^,^55^ should make this an achievable goal. Based on tracks near predated plover and tern nests at our field site, we can infer that mammals are important clutch predators (Küpper & Cruz-Lopez, unpublished data). Crested Caracaras and other birds may also be significant predators. Importantly, avian and reptilian predators have been reported elsewhere for Snowy Plovers^56^,^57^,^58^ and Least Terns^55^. However, identifying only the local predators at Bahía de Ceuta may not be enough. Snowy Plovers are highly mobile during the breeding season. Genetic and resighting data demonstrate that particularly polyandrous plover females move regularly move large distances between breeding attempts^59^,^60^. Therefore, each female likely encounters different nest predator communities during a single breeding season. Egg camouflage has likely evolved in response to many diverse predators – and not necessarily just in response to locally present predators. Experiments could test whether egg appearance has changed over time due to relaxed selection (*sensu* Lahti^61^), particularly if there are locations where eggs were once exposed to a diverse suite of predators but are now just exposed to one (*i*.*e*., remote island populations). Discovering the main predators of Snowy Plover and Least Tern nests is also an important priority for conservationists^14^,^58^. The “near threatened” Snowy Plovers can lose a very large proportion of nests to predation each year^58^. This is particularly important for the breeding population at Bahía de Ceuta, which has declined severely since 2006.

To model camouflage from the relevant predator perspective using digital images, one must use a specially calibrated camera that meets two requirements: 1) the spectral sensitivities of the camera’s sensors are known, and 2) the camera’s sensors capture the full range of visible wavelengths for the relevant predator^36^,^38^. In this study, we did not use a calibrated full-spectrum ultraviolet-sensitive camera, nor did we characterize the full suite of egg predators. For these reasons, we elected to use the CIELAB space, which yields device-independent measures, permits comparison to other recent studies of egg camouflage (Lovell *et al.*^24^), and provides a first approximation of camouflage metrics. Our approach, which includes using standard color charts to calibrate images in the human visible range^35^, may be a good first step for researchers lacking a full-spectrum camera. In future studies, it will be essential to determine the relevant egg predators in this system and to apply a broad range of species-specific visual models^38^. This is more straightforward for assessments of color, since the spectral sensitivities of cone-types for many different species are available. Much less is known about variation in edge detection mechanisms and pattern perception across species^62^.

A second goal for future work will be to evaluate the relative importance of different anti-predator defenses at the nest. Egg camouflage is likely one of many defenses employed by plovers and terns: in reality, the behavior and appearance of incubating parents can also have a large effect on predation^63^. Many ground-nesting birds have also evolved camouflaged plumage^64^ or – in some cases – camouflaged smells via seasonal modification of their preen wax^65^. Ultimately, egg appearance is likely to be a compromise between many competing forces. Perfectly camouflaged eggs are unlikely to evolve because the egg phenotype is pulled in many directions, not just in response to the diverse visual systems of predators but also in response to other demands (*e*.*g*., thermoregulation, antimicrobial defense, eggshell strength, sexual signaling)^7^.

In conclusion, using objective measures of visual camouflage is an important first step to understanding which features of eggs and the background substrate may be effective in preventing predator detection. Our assessment of egg camouflage by Snowy Plovers and Least Terns suggests a number of species differences. Our EdgeDetector code - which builds on quantitative approaches proposed and used elsewhere^24^,^25^ - makes it easy to quantify aspects of camouflage related to edge and outline detection and provides researchers with an unbiased approach for objective camouflage description. More broadly, tools that relate camouflage to the potential perceptual properties of predators move us one step closer to understanding whether there are some universal principles of animal camouflage^26^.

## Additional Information

**How to cite this article**: Stoddard, M. C. *et al.* Camouflage and Clutch Survival in Plovers and Terns. *Sci. Rep.* **6**, 32059; doi: 10.1038/srep32059 (2016).

## Supplementary Material

Supplementary Information

## Figures and Tables

**Table 1:** Camouflage characteristics of Least Tern and Snowy Plover clutches at Bahía de Ceuta.

Camouflage mechanism	Variables	Least Tern (N = 24)	Snowy Plover (N = 30)	t	p
a) Pattern complexity	BgEdge	0.07 ± 0.01	***0***.***08*** ± ***0***.***01***	2.2	**0**.**03**
	EggEdge	0.05 ± 0.01	0.08 ± 0.02	7.1	**<0**.**001**
	CompRat	0.8 ± 0.2	1.1 ± 0.3	3.3	**<0**.01*
b) Disruptive effects	ContEdge	***0***.***08*** ± ***0***.***006***	0.09 ± 0.009	6.5	**<0**.**001**
	DisRat	1.7 ± 0.6	***1***.***3*** ± ***0***.***7***	5.1	**<0**.**001**
	VisRat	1.3 ± 0.3	1.3 ± 0.2	−0.2	0.81
c) Egg and substrate color matching	DeltaE	22.2 ± 4.9	21.0 ± 4.3	1.0	0.33
	PropBgToEggColorMatch	0.3 ± 0.2	***0***.***5*** ± ***0***.***2***	4.8	**<0**.**001**

Significant differences are in bold. Better camouflage is shown in italic bold font. No specific effect of EggEdge on camouflage is predicted.

^*^Least Tern and Snowy Plover clutches differ in CompRat but these differences are not significantly different from 1, meaning that neither species is better camouflaged.

**Table 2:** Model selection for variables associated with survival of 30 Snowy Plover and 24 Least Tern nests at Bahía de Ceuta (df = degrees of freedom; logLik = log likelihood; AICc = AIC values adjusted for small samples; ∆_i_ = Delta AICc; ω_i_ = model weight).

Model	df	logLik	AICc	∆_i_	ω_i_
Species	1	−65.26	132.6	0.00	0.17
Species + DisRat	2	−64.59	133.4	0.82	0.11
Species + CompRat^2	2	−64.78	133.8	1.2	0.09
Species * DisRat	3	−63.69	133.8	1.25	0.09
Species + PropBgToEggColorMatch	2	−64.81	133.9	1.26	0.09
Species * PropBgToEggColorMatch	3	−63.78	134	1.45	0.08
Species + VisRat	2	−65.23	134.7	2.1	0.06
Species + DeltaE	2	−65.23	134.7	2.11	0.06
Species + PropBgToEggColorMatch + DisRat	3	−74.38	135.2	2.65	0.05
Species * CompRat^2	3	−64.74	136	3.37	0.03
Species * VisRat	3	−64.96	136.4	3.81	0.03
DisRat	1	−67.2	136.5	3.89	0.02
DeltaE	1	−67.26	136.6	4.01	0.02
PropBgToEggColorMatch	1	−67.27	136.6	4.02	0.02
Species * DeltaE	3	−65.13	136.7	4.15	0.02
CompRat^2	1	−67.38	136.8	4.24	0.02
VisRat	1	−67.45	137	4.38	0.02
Species + PropBgToEggColorMatch + DeltaE + CompRat^2 + VisRat + DisRat	6	−63.68	141.1	8.55	0
PropBgToEggColorMatch + DeltaE + CompRat^2 + VisRat + DisRat	5	−66.82	144.9	12.3	0
