# Supplementary material for: Camouflage and Clutch Survival in Plovers and Terns
Source: Sci Rep. 2016 Sep 12;6:32059. doi: 10.1038/srep32059 (PMC5018847; doi:10.1038/srep32059)

Supplementary Information

Stoddard, M. C. et al. Camouflage and Clutch Survival in Plovers and Terns. Sci. Rep. 6, 32059; doi: 10.1038/srep32059 (2016).

Camouflage and Clutch Survival in Plovers and Terns

Mary Caswell Stoddard[1,2], Krisztina Kupán[3], Harold N. Eyster[2], Wendoly Rojas-Abreu[4], Medardo Cruz-López[5], Martín Alejandro Serrano-Meneses[4,6] & Clemens Küpper[3]

[1]Department of Ecology and Evolutionary Biology, Princeton University, Princeton, NJ 08544, USA. [2]Museum of Comparative Zoology, Department of Organismic and Evolutionary Biology, Harvard University, Cambridge, MA 02138, USA. [3]Institute of Zoology, University of Graz, Universitätsplatz 2, 8010 Graz, Austria. [4]Laboratorio de Biología Evolutiva, Centro Tlaxcala de Biología de la Conducta, Universidad Autónoma de Tlaxcala, Carretera Tlaxcala-Puebla km 1.5, 90070, Tlaxcala, México. [5]Posgrado en Ciencias del Mar y Limnología, Universidad Nacional Autónoma de México, Unidad Académica Mazatlán, Av. Joel Montes Camarena S/N, 82040, Mazatlán, Sinaloa, México. [6]Posgrado en Ciencias Biológicas, Universidad Autónoma de Tlaxcala, México

Correspondence and requests for materials should be addressed to M.C.S. (email: mstoddard@ princeton.edu)

Table S1. Egg color and pattern characteristics of Least Tern and Snowy Plover clutches. Significant differences are in bold.

**Variable**		**Least Tern (N=24)**	**Snowy Plover (N=30)**	**t**	**p**
Mean Lab of the eggs	L	58.1 ± 7.5	55.4 ± 7.7	-1.3	0.20
	a	3.2 ± 1.6	2.4 ± 1.2	-2.2	**0.04**
	b	12.0 ± 2.9	12.5 ± 2.4	0.6	0.55
Lab of the egg background only	L	63.7 ± 7.7	60.5 ± 8.3	-1.4	0.16
	a	2.3 ± 1.8	1.7 ± 1.3	-1.7	0.10
	b	12.5 ± 3.3	13.4 ± 3.1	1.0	0.31
Lab the egg maculation only	L	43.8 ± 9.1	38.7 ± 11.3	-1.8	0.08
	a	5.3 ± 1.9	4.7 ± 2.3	-1.4	0.18
	b	10.5 ± 2.4	9.2 ± 2.9	-1.8	0.07
Proportion of maculation		0.3 ± 0.2	0.3 ± 0.1	-1.2	0.24

Figure S1. Average reflectance spectra for Snowy Plover (n = 12 eggs from 4 clutches) and Least Tern eggs (n = 6 eggs from 3 clutches).

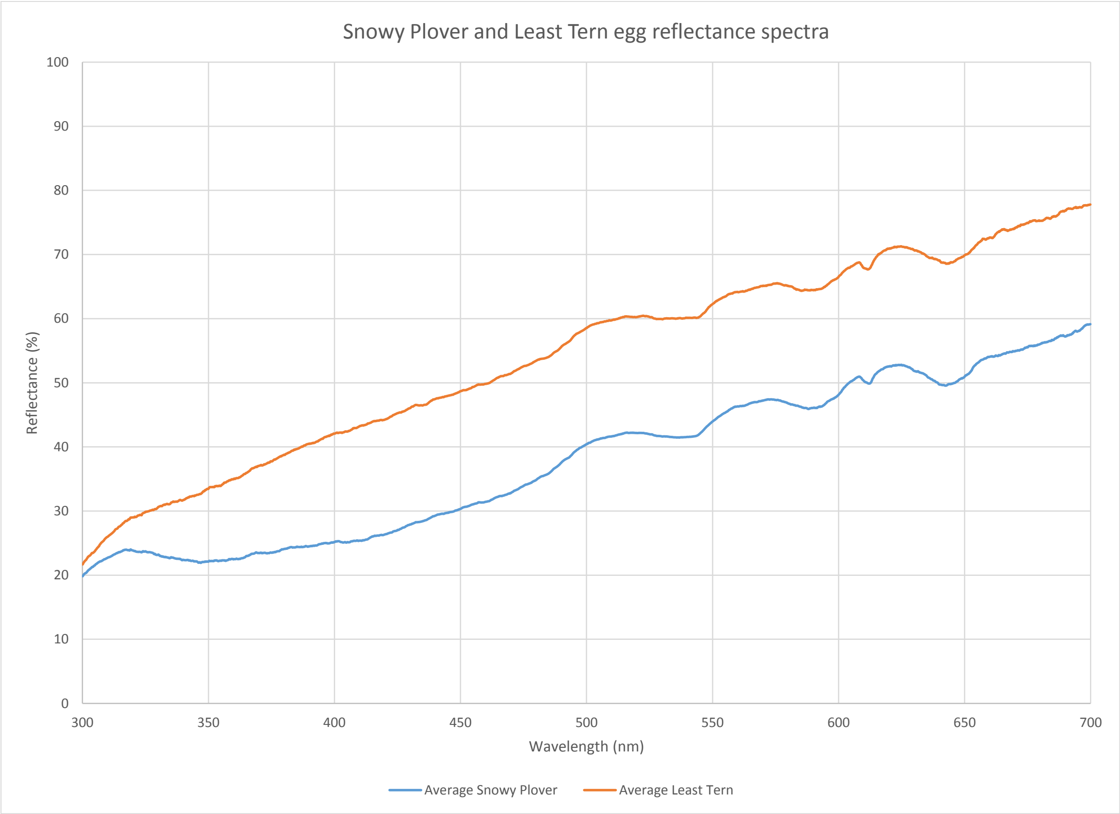

Supplement: Supplementary Information [file srep32059-s1.pdf]
